# Seawater Desalination by Modified Membrane Distillation: Effect of Hydrophilic Surface Modifying Macromolecules Addition into PVDF Hollow Fiber Membrane

**DOI:** 10.3390/membranes11120924

**Published:** 2021-11-25

**Authors:** Mochammad Purwanto, Nindita Cahya Kusuma, Ma’rup Ali Sudrajat, Juhana Jaafar, Atikah Mohd Nasir, Mohd Haiqal Abd Aziz, Mohd Hafiz Dzarfan Othman, Mukhlis A Rahman, Yanuardi Raharjo, Nurul Widiastuti

**Affiliations:** 1 Department of Chemical Engineering, Institut Teknologi Kalimantan, Balikpapan 76127, Indonesia; m.purwanto@lecturer.itk.ac.id (M.P.); nindita.ck@gmail.com (N.C.K.); marupali10@gmail.com (M.A.S.); 2 Advanced Membrane Technology Research Centre (AMTEC), Universiti Teknologi Malaysia, Skudai 81310, Malaysia; atikah.mohdnasir@utm.my (A.M.N.); haiqalabdaziz@gmail.com (M.H.A.A.); hafiz@petroleum.utm.my (M.H.D.O.); r-mukhlis@utm.my (M.A.R.); 3 Membrane Science and Technology Research Group, Chemistry Department, Faculty of Science and Technology, Universitas Airlangga, Surabaya 60115, Indonesia; yanuardiraharjo@fst.unair.ac.id; 4 Department of Chemistry, Institut Teknologi Sepuluh Nopember, Surabaya 60111, Indonesia; nurul_widiastuti@chem.its.ac.id

**Keywords:** membrane distillation, polyvinylidene fluoride, surface-modifying macromolecules, seawater desalination

## Abstract

Hollow fiber membranes of polyvinylidene fluoride (PVDF) were prepared by incorporating varying concentrations of hydrophilic surface-modifying macromolecules (LSMM) and a constant amount of polyethylene glycol (PEG) additives. The membranes were fabricated by the dry-wet spinning technique. The prepared hollow fiber membranes were dip-coated by hydrophobic surface-modifying macromolecules (BSMM) as the final step fabrication. The additives combination is aimed to produce hollow fiber membranes with high flux permeation and high salt rejection in the matter of seawater desalination application. This study prepares hollow fiber membranes from the formulation of 18 wt. % of PVDF mixed with 5 wt. % of PEG and 3, 4, and 5 wt. % of LSMM. The membranes are then dip-coated with 1 wt. % of BSMM. The effect of LSMM loading on hydrophobicity, morphology, average pore size, surface porosity, and membrane performance is investigated. Coating modification on LSMM membranes showed an increase in contact angle up to 57% of pure, unmodified PVDF/PEG membranes, which made the fabricated membranes at least passable when hydrophobicity was considered as one main characteristic. Furthermore, The PVDF/PEG/4LSMM-BSMM membrane exhibits 161 °C of melting point as characterized by the DSC. This value indicates an improvement of thermal behavior shows so as the fabricated membranes are desirable for membrane distillation operation conditions range. Based on the results, it can be concluded that PVDF/PEG membranes with the use of LSMM and BSMM combination could enhance the permeate flux up to 81.32 kg·m^−2^·h^−1^ at the maximum, with stable salt rejection around 99.9%, and these are found to be potential for seawater desalination application.

## 1. Introduction

Water shortage is one of the most critical issues that need to be overcome properly since the majority of mankind’s daily activities are water-dependent. The increase in the human population reflects much need for a clean water supply [1]. On the other hand, clean water sources from nature do not become replenished to accommodate the population’s water usage needs, which also continuously produce wastewater. This causes unfair distribution of clean water, and it affects many people around the world, especially in developing countries such as the Middle East, Africa, Asia, and Latin America, which experienced a lack of access to potable water and faced obstacles to meet all-year-round irrigation in the agricultural sector [2]. Moreover, existing water treatment processes using other than MD are still requiring high energy to be operated, which is something that needs to be avoided due to lower efficiency and higher operating cost [3]. Hybridizing the synergy between innovative and low energy-driven technology of MD and infinite water resource such as seawater is a highly potential solution toward having sustainable water reclamation technology.

MD process may be used as a substitute for conventional desalination process, for example, reverse osmosis (RO), which is commercially used these days [1,4,5]. There are some advantages of using the MD process rather than the RO process. The advantages are MD process produces distillates purity 30 times higher than the RO process, the MD process operates at a lower pressure, which saves more energy consumption rather than the RO process, and the hydrophobic characteristic of the MD membrane performs lower fouling and concentration polarization [6]. By selecting the appropriate membrane material with hydrophobic properties, MD is able to reduce the chemical interactions between membranes and the feed solution, thus manageable to attract clean water permeation.

From the design and configuration point of view, MD is more compact as compared to an RO system [7]. MD has a few configurations, such as direct contact membrane distillation (DCMD), air gap membrane distillation (AGMD), vacuum membrane distillation (VMD), and sweeping gas membrane distillation (SGMD). DCMD is the most studied configuration in laboratory-scale because of its simple structure, easiness in operation, and has been proved could provide a higher permeate flux relative to AGMD and SGMD due to immediate contact of the membrane itself with both streams, supply, and permeate, which indicates minimum mass transfer resistance [3,8].

All this time, membrane hydrophobicity has become a specific concern in MD study. The hydrophobic property of the membrane prevents liquid penetration through the membrane. The more hydrophobic material, the more selective membrane is. Polytetrafluoroethylene (PTFE), polypropylene (PP), and polyvinylidene fluoride (PVDF) are suggested materials for MD fabrication because of their hydrophobic nature characteristic. PVDF is the one among all mentioned hydrophobic materials that possess suitable stability in thermal and suitable resistance of chemical [3,5]. This fluoro-typed polymer is also flexible, easy to process, and has excellent mechanical properties. To further enhance the competitiveness of PVDF membrane in waste and wastewater treatment applications, surface modification was found to be a promising solution.

The hydrophobicity/hydrophilicity properties of PVDF can be modified by incorporating additives such as surface-modifying macromolecules (SMM). SMM is a polymer available in hydrophobic and hydrophilic structures depending on the functional group attached at the end of the polymer tail. The SMM that has hydrophobic nature, which is structurally ended up with fluorohydrocarbon group [9], is abbreviated as BSMM, while hydrophilic SMM with hydroxyl function end-group [10] is shortened as LSMM.

Both kinds of SMMs are designed to be used as hollow fiber membranes additives. As the concept of the MD process, only the volatile component in the vapor phase will pass through the porous membrane, and it will be condensate then become liquid permeate [11]. On the other hand, the retentate, which is a non-volatile component in the liquid phase, will be retained at the feed side and will be circulated continuously [3,11]. The purpose of integrating LSMM in the permeate side of an MD membrane is theoretically could provide higher flux permeation by creating a hydrophilic condition in the lumen side (for hollow fiber) out-in with co-current flow configuration or upper layer (for flat sheet) membrane as the vapor will flow up from bottom layer that flowed by hot feed solution [12]. That setup scheme of configuration will consequently spark a higher permeation. The use of LSMM gives several advantages, such as it can increase the viscosity of dope solution in which further affects the thickness and compactness of the prepared membrane [13]. Moreover, by incorporating variations of LSMM, this study will find out the limitation amount in using LSMM to prevent the possibility of a wetting phenomenon.

In our previous work [14], the highlight on the impact of BSMM addition to the hydrophobicity property of the PVDF/LSMM/BSMM membranes for mitigating the membrane’s pore wetting issue has been successfully investigated. It gives better performance in blocking the retentate (selectivity). As Khayet and Essalhi [15] mentioned that modified PVDF membrane using BSMM resulted in smaller membrane pore size, which affects flux performance; thus, additional additive is needed. The larger pore size can be formed by using PEG (polyethylene glycol) during the fabrication process, where it gave an influence in membrane morphology structure, which includes pore size diameter and porosity according to the desired sizes for MD application [16]. Previous research also reported the formation of bridge complexes between glycerol, polymer fluorine, and solvent, which deteriorated the polymer chains flexibility and caused a decrease in distributive freedom of the polymer in the dope solution [16]; thus, typical pores were shaped.

The objective of this study, therefore, was to investigate the effect of LSMMs blending with PVDF/PEG as base material to be performed in seawater desalination. This paper not only explores morphological characteristics of the modified membrane but also assesses its nature surface via contact angle measurement and MD permeation test.

## 2. Methodology

### 2.1. Materials

Polyvinylidene fluoride (PVDF) polymer (Kynar^®^ 760) pellets purchased from Arkema Inc., Philadelphia, PA, USA, were used as the main membrane-forming material. The molecular weight (MW) of PVDF is 440.000 g/mol. Hydrophobic surface-modifying macromolecules (BSMM) with MW of 27,100 g/mol and hydrophilic surface-modifying macromolecules (LSMM) with MW of 4050 g/mol were used as additives and were obtained from the colleague from the University of Jordan (Amman, Jordan). N,N-dimethylacetamide (DMAc) (Merck, >99%) was used as the solvent to dissolve PVDF and LSMM. Polyethylene glycol (PEG) with MW of 400 g/mol was purchased from Sigma Chemical. N-Methyl-2-pyrrolidone (NMP, AR Grade) from RCI Labscan Limited (Bangkok, Thailand) and Ethanol (99.9%) were used as solvents for coating agent solution. The distilled water was used as the internal coagulant, while tap water was used as an external coagulant.

**Figure 1:**
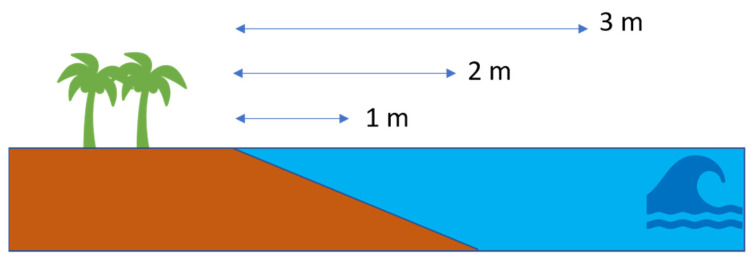
Seawater sampling at Pontian seaside.

Samplings for seawater samples were conducted in Pontian, Johor, Malaysia, and involved three sampling locations of Pontian seaside. Seawater samples were collected in triplicate from 1, 2, and 3 m away from the bay, as depicted in Figure 1. According to the variations of LSMM content, the 3 wt. % of LSMM was simulated using 1 m sample of seawater, while 4 and 5 wt. % of LSMM membranes were used 2 and 3 m of seawater samples, respectively.

### 2.2. Preparation of PVDF/PEG/LSMM Hollow Fiber Membrane

The membranes were prepared using a set of spinning equipment through non-solvent-induced phase separation (NIPS). The process was started by making spinning dopes after the PVDF pellets dried to reduce water content. The LSMM was dissolved in DMAc solvent with the determined amount shown in Table 1. The dissolution was then followed by PVDF addition and continued by stirring them at 60 °C for 24 h. PEG was then added into the dope and continuously stirred for 2 h until the solution became more homogeneous. This step is important in order to eliminate the air bubble trapped in the dope solution; an ultrasonic bath was subsequently used for 12 h at a constant temperature of 60 °C.

As the solution was degassed, it was then fed into the annulus of the spinneret with the help of a gear pump; meanwhile, the bore fluid (distilled water) was passed through the inner tube of the spinneret with the help of a peristaltic pump. The produced hollow fiber membranes were taken up by a rotating drum and immersed in a bath of tap water for 72 h to remove residual solvent. At the final step, the membranes were dried for at least 3 days until ready to use [17].

The details of the spinning process condition applied in this study are shown in Table 2.

For the surface modification process by dip-coating method, one end side of PVDF/PEG/LSMM hollow fibers was corked using epoxy to hinder the entry of coating solution into the lumen side. As the coating solution was prepared by dissolving 1 wt. % of BSMM into a mixed solvent of 5 wt. % NMP and 95 wt. % Ethanol, the membranes were then dipped into coating solution for 30 s and dried in ambient air for a day.

### 2.3. Membrane Characterization

#### 2.3.1. Water Contact Angle (WCA)

The water contact angle measurement was conducted by using a set of goniometers (Kruss Gambult, Germany). A sessile drop method was used to measure the contact angle seen from the fiber horizontal surface. A total of 1 µL of water droplet was introduced on the surface of the fiber, and the image profile of the drop was shown by the software.

#### 2.3.2. Field Emission Scanning Electron Microscope (FESEM)

The FESEM (Hitachi, model: TM3000 tabletop microscope, Tokyo, Japan) was used to observe membrane morphology by its surface and cross-sectional area. The image result is then analyzed using ImageJ software (Java^TM^ Platform SE binary) to observe pore diameter and porosity. The FESEM images were turned into white regions, which are represented as particles, and black regions, which are represented as pores. The porosity of the hollow fiber membrane was calculated from the percentage of black pixels to total pixels. The average area as pore diameter from the FESEM image was estimated by assuming the porous cylindrical texture of the membrane [18].

#### 2.3.3. Differential Scanning Calorimetry (DSC)

The thermal stability of the fabricated membranes was examined using a differential scanning calorimetry (DSC) set of instruments (Mettler Toledo, SDTA851, Columbus, OH, USA). The sample was tested with the range of temperature 0–250 °C in 22 min.

### 2.4. Membrane Performance

#### 2.4.1. Permeate Flux

**Figure 2:**
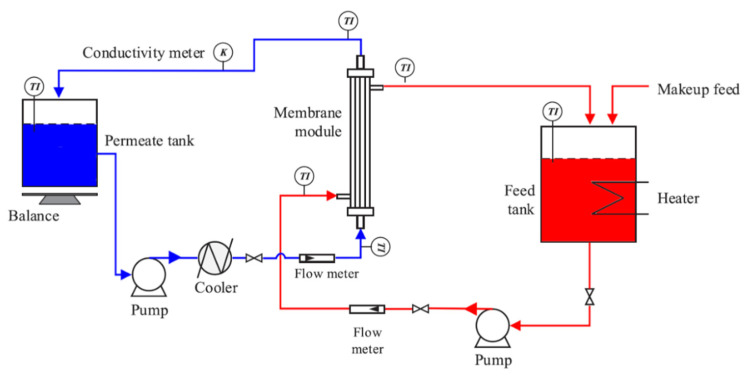
Schematic view of the installed DCMD apparatus.

The measurement of permeate flux was started by assembling a DCMD apparatus scheme shown in Figure 2 in which feed as a hot solution was designed to flow through the shell side while the permeate as a cold solution was flowed past through the lumen side of the membranes.

The feed consisted of 3.5 wt. % of NaCl (salt) was stored at a feed tank, and the temperature was monitored at 70 °C by using a temperature indicator (TI), whereas the cold water at permeate tank is controlled to be 20 °C. Both hot and cold solution rate was 0.5 m/s and flowed co-currently through the module. To calculate the permeate flux, the following formula was used:

J = D/(A × Δt)(1)

where J is pure water flux (kg·m^−2^·h^−1^), D is permeate amount (kg), A is effective membrane surface area, and Δt is sampling time (h).

#### 2.4.2. Salt Rejection

As MD was purposed for the seawater desalination process, membrane performance in terms of salt rejection needs to be concerned. The data collection involves the use of a conductivity meter to measure salinity as a parameter for salt concentrations. The percentage of rejection was calculated as follows:

R = (Cf − Cd)/Cf × 100%(2)

where R is salt rejection (%), Cf is salt concentration in feed solution (µS), and Cd is salt concentration in permeate (µS).

## 3. Results and Discussion

### 3.1. Water Contact Angle of PVDF/PEG/LSMM/BSMM Membranes

**Figure 3:**
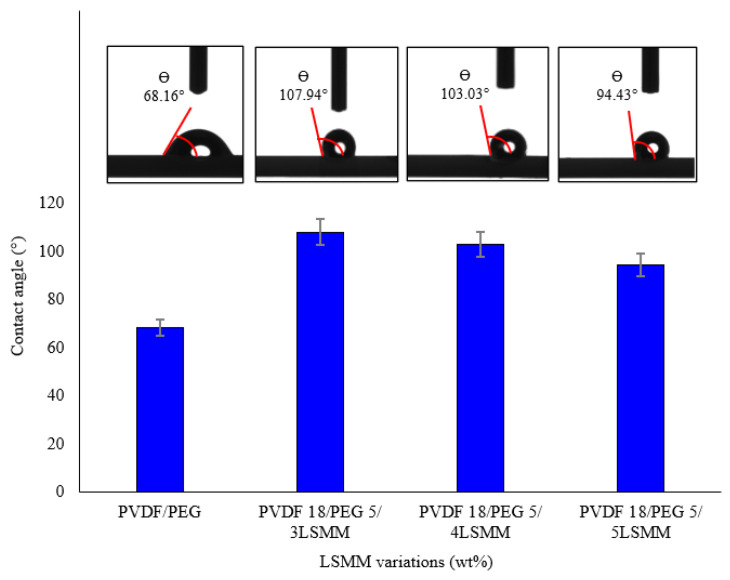
Water contact angle measurement result.

Contact angle measurement is a common method to identify the hydrophobicity/hydrophilicity properties of a material surface. Figure 3 shows the contact angle result of the fabricated membranes. The surface energy and surface tension of the liquid are a few factors that influence contact angle measurement [19], in which pure PVDF material commonly has a surface energy of 30.3 mJ·m^−2^ [7].

The surface energy will be affected by the contact angle. The smaller surface energy produced, the greater contact angle obtained [9]. This is conducted by adding a BSMM coating agent to produce low surface energy. The use of hydrophilic agent LSMM, mixed in membrane preparation for MD desalination application. As the MD process requires hydrophobic characteristics to allow vapor only to pass the pores, the fabricated modified membranes reached the minimum hydrophobicity value for MD, which is more than 90° [20,21]. According to previous research that resulted in a contact angle of 55° for PVDF 18/PEG 5/4LSMM [13], BSMM coating was added to increase the contact angle of the membrane and create the contact angle higher than 90°. The increase in contact angle due to the presence of fluorine atoms in the BSMM creates low surface tension and makes a hydrophobic state on the membrane [9]. It is supported by the presence of BSMM on the outer surface of the hollow fiber membrane so as to prevent excess wettability [22].

**Figure 4:**
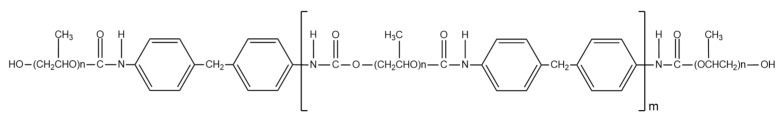
Chemical structure of LSMM.

With the same amount of coating agent of BSMM, it indicated a slight reduction in contact angle value upon the addition of LSMM. This corresponds to the chemical structure of LSMM shown in Figure 4, which is end-capped by an OH bond that is attractive to the water molecules due to its polarity nature [23].

As shown on Figure 3, the contact angle resulted a slight difference between LSMM membrane variations, this insignificant change in contact angle value is deemed to provide no effect on the MD performance of the membranes.

### 3.2. Characteristics Study of Modified PVDF Membrane

#### 3.2.1. Morphological Structure

**Figure 5:**
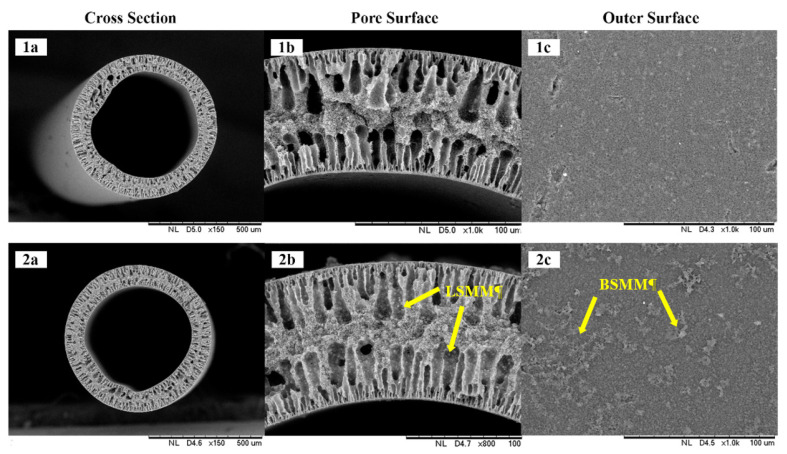
FESEM pictures of the prepared membranes: (**1**) PVDF/PEG, (**2**) PVDF/PEG/3LSMM-BSMM 1 in which (**a**) exhibits cross section view, (**b**) exhibits pore surface, (**c**) exhibits outer surface.

Figure 5 shows the cross-sectional view of fabricated modified PVDF membranes. As can be clearly seen, the membrane morphology had two layers of finger-like structures, which is a typical structure for PVDF by incorporating PEG additive [5,20]. The LSMM that has been mixed during the PVDF/PEG/LSMM dope solution preparation [21] is successfully dispersed, shown by Figure 5(2b), which exhibits the presence of white particles on the pore surface. On the other hand, the BSMM on the membrane surface, as shown in Figure 5(2c), is purposed to balance the hydrophobicity. The surface energy of BSMM due to the presence of fluorine atoms resists interactions with the oxygen atoms in water molecules [24]. The hydrophobic features then limit the wettability phenomena.

With respect to membrane physical characteristics, it is found that LSMM played a role in increasing membrane thickness (Table 3). Membrane thickness impacts both mass and heat transfer in a different correlation when implemented in the MD process. Membrane thickness is inversely proportional to the permeate flux, where an increase in thickness has a negative effect, and lower flux occurs due to its role as a mass transfer resistance [3,9,25], and vice versa. This study obtained the thickness of the membrane was increased from 165 to 183 µm in which is still in the range of common commercial membrane for MD that ranged from 40 to 250 µm [7]. Aside from the membrane effect on flux permeation, the thickness also reflects the mechanical strength of the membrane [22]. The higher polymeric content increases casting solution viscosity; thus, the molecular movement is restricted, and it is difficult to form more pores in the membrane. Then, the membrane became thicker and strengthened the mechanical property. As studied by Zhang et al., the tensile strength was examined to evaluate PVDF membrane samples, and it resulted in a rising trend of tensile strength as the thickness increased [22]. Mechanical strength is important to be concerned because it further underlies deformation mechanisms that are critical not only for membrane structure design but also for their reliability and lifetime prediction [26]. In contrast, the addition of LSMM decreased the membrane pore size. This result was aligned with previous research, which mentioned a similar trend [23]. As SMM would migrate to the interface of the membrane and air, the LSMM is then distributed along the pore surface, thereby reducing the pore size even if the micro-scale changes. However, it still reaches the minimum requirement of pore size of the MD process, which is 0.20 µm. A smaller pore size is needed to avoid wetting on the membrane or the excess penetration of feed into the membrane [4,7].

The thickness of the hollow fiber membrane was analyzed by using FESEM images and a set of FESEM instruments that can measure micro size. The measurement is conducted by subtracting the outer diameter from the inner diameter of the hollow fiber on the micron scale. The difference between those is then called thickness.

**Figure 6:**
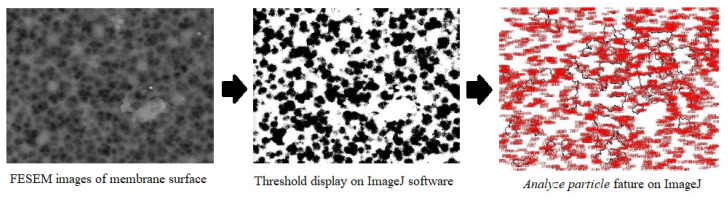
Pore size and surface porosity analysis using ImageJ.

Another character of membrane structure is porosity, which was simultaneously analyzed with pore size measurement using ImageJ software, as exhibited in Figure 6. The software counted the percentage of the void area (black holes) that reflected porosity and pores through average pore size. The software counts the hole as red numbers distributed in Figure 6. The voids were counted by the software show the average pore diameter in microns and the percentage of them that reflect the surface porosity. As porosity is also related to the permeation performance, the desired porosity in the membrane structure is as high as possible to maximize the permeation because it reflects the capacity for the membrane to allow vapor to flow into the inner side of the membrane. The larger percentage of porosity, the higher permeate flux obtained [4]. However, membranes with high porosity have low mechanical strength, so they are prone to leakage or damage to the membrane.

Therefore, ideal porosity is considered to meet MD specifications, which is 85% at the maximum. As the porosity aspect influences permeation value, this study used a PEG additive to maintain the optimum porosity of the membrane [27]. Furthermore, PEG worked as an anti-fouling and anti-bacterial material, which gives a longer membrane lifetime [28].

#### 3.2.2. Thermal Stability

MD process requires a high temperature of feed to be operated, which was varied in the range of 60–85 °C [27]. The thermal property of the membrane was considered to be evaluated. This study used differential scanning calorimetry (DSC) as a thermoanalytical technique in which the heat flow rate difference into a sample and a reference is measured as a function of temperature [28]. The test resulted in 161 °C of melting point for PVDF/PEG/3LSMM membrane. It was such an improvement of a previous study by Hou et al., which exhibited the melting point of 150 °C for PVDF/PEG membrane [5]. The use of LSMM increased the molecular weight of the membrane material, which influenced the melting point [22,29].

**Figure 7:**
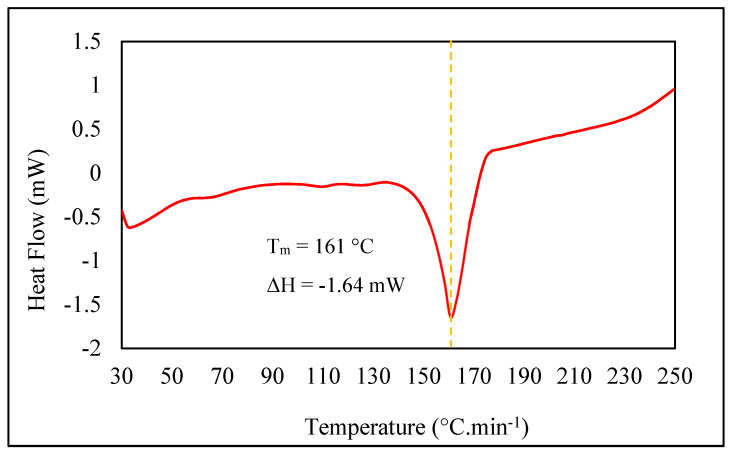
Thermogram curve as DSC result.

A melting endothermic peak (Tm) on the thermogram curve shown in Figure 7 indicates the temperature at which the solid material melts [30]. It can be interpreted that the fabricated membrane of this study was likely to be implemented to the MD process in terms of its thermal strength, as the MD operations are constantly operating in a range of 60–90 °C [7]. In addition, because the measurement result reflects the thermal resistance during MD operations, thus the membrane tends to be more stable of hot feedwater.

### 3.3. DCMD Performance

**Figure 8:**
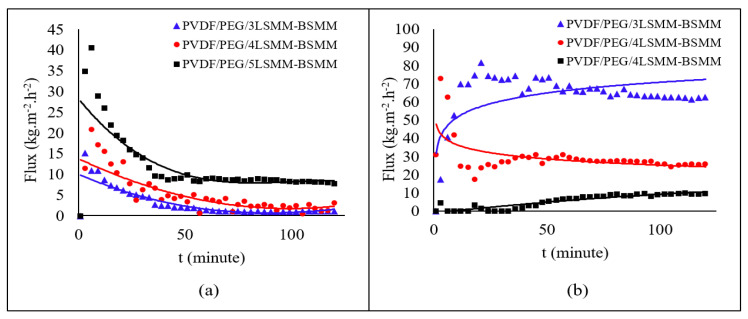
Membrane flux permeation of (**a**) 3.5 wt. % saltwater and (**b**) seawater in the desalination process.

DCMD performance in terms of permeation flux and salt rejection were obtained for modified membrane vary with the LSMM content percentage are shown in Figure 8. The desalination process was conducted using 3.5 wt. % of NaCl solution and seawater in a separate operation. The results of the DCMD process are shown in Figure 8. In using NaCl solution as feed water, PVDF/PEG/5LSMM-BSMM membranes had the highest permeate flux value of 40.53 kg·m^−2^·h^−1^. Other variations of LSMM content gave similar trends if it was simulated using NaCl solution, in which the membranes performed stable fluxes after the first 50 min.

On the contrary, PVDF/PEG/5LSMM-BSMM was performing the lowest flux if it was tested using seawater within 2 h durations. Indeed, PVDF/PEG/3LSMM-BSMM had time to reach 81.32 kg·m^−2^·h^−1^. Nevertheless, there would be some concerns in reviewing this membrane due to its odd trendline compared to others. The wetting phenomenon can be one issue when assessing the uncommon trend of flux permeation. This is supported by the lowest salt rejection resulting from the membrane, which will be discussed in more detail in the next section. The wetting phenomenon is possibly caused by membrane damage due to impurities brought by feed water, as seawater has much content other than salt itself. Those impurities can be destructive to the membrane structure, thereby reducing the life of the membrane during operation. For instance, the chlorine content that still passes the feed water pre-treatment, in which the chlorine content must be lower than 1 ppm to proceed into the polymeric membrane [31].

As the characteristic of LSMM material is hydrophilic, whereas common MD requires a more hydrophobic nature [32], the identification of the optimum amount of LSMM incorporation is desirable toward achieving high flux and high rejection simultaneously. Among all variations, membranes with 4 wt. % of LSMM had a typical trend of flux when simulated using both types of feedwaters. Hence, the membrane exhibited the most consistent performance. In accordance with membrane structure, PVDF/PEG/4LSMM-BSMM was the thinnest fiber that potentially exhibits higher flux, as well as had the smallest porosity that may controllably vapor flow. Even though the trendline of seawater desalination continued to decrease slightly due to the deposition of salts or other impurities on the membrane surface over time, it would result in a reduction in the effective membrane surface, which further affect permeate flux [18]. PVDF/PEG/4LSMM-BSMM membranes performed a maximum flux of 20.74 kg·m^−2^·h^−1^, which is such an improvement from the previous study that researched PVDF-based membrane for saltwater desalination resulting in 19.58 [30] and 6.8 kg·m^−2^·h^−1^ [5].

On the other hand, the overall salt rejection examination resulted in an increasing trend in the graph. Among these LSMM content variations, 5 wt. % of LSMM content membranes showed a superior separation factor of 99.95% due to the smaller pore size the membranes had. Membranes with salt rejection above 99% can be considered as a promising membrane for MD [33]. In addition, this can be caused by the hydrophilic property owned by LSMM that can minimize the potential of fouling and affect the improvement of salt rejection performance. Several previous studies [13,34] have revealed that increasing the membrane hydrophilicity can effectively reduce the membrane fouling as the use of LSMM can overcome the salt or other mineral deposits, which is followed by longer life-operation during the desalination process.

**Figure 9:**
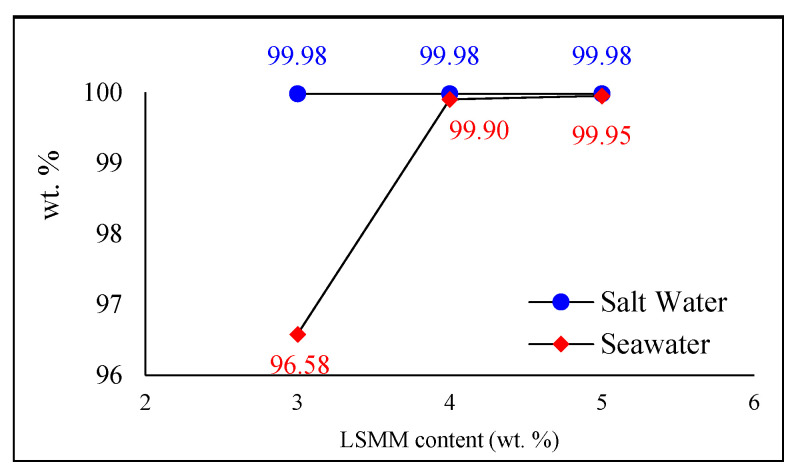
Salt rejection performance.

According to Figure 9, the 3 wt. % of LSMM membrane results in the smallest salt rejection due to the presence of organic contamination in the seawater, causing fouling and wetting of the membrane.

As researched by Nalley et al. [35], the water quality is decreasing closer to land in which water at coastal areas tends to contain higher contaminants compared to deeper spots. This leads to the increase in water conductivity, and thus, the membrane performs a low salt rejection. Above all, 3 and 4 wt. % of modified membranes that have similar characterizations can be considered as two potential optimum compositions of modified membranes through the LSMM and BSMM incorporation. By concerning other factors such as fabrication operation conditions [36] to create an optimum morphological structure that is comprised of thickness, porosity, and pore size diameter. This then leads to the correlation between these optimum amounts of LSMM as the discovery of this research, and morphological characterization, which is suggested to further research for advanced seawater desalination application.

## 4. Conclusions

In this study, the effect of incorporation of LSMM in PVDF/PEG hollow fiber membrane coated by BSMM was investigated. The membranes were evaluated by physical, structural, and MD process tests. The characterization results that the PVDF/PEG/LSMM-BSMM hollow fiber membranes had two layers of finger-like structure morphology and tended to be hydrophilic with water contact angle results variated from 94.43° to 107.94°. The hollow fiber membrane’s thermal property showed that adding both LSMM affected on membrane melting point. The modification membrane has a melting point around 161 °C, which is higher than the previous study about unmodified (PVDF/PEG) membranes. By introducing a dual-layered membrane comprising of hydrophobic and hydrophilic properties at the outer and inner layers of the PVDF membranes has indeed improved the salt rejection and flux, respectively. This study revealed that LSMM loadings at 4 wt. % were the best membrane for MD in order to achieve stable results in both flux and salt rejection performance. However, the fabricated membranes in 3 wt. % of LSMM loadings can be considered as a promising membrane due to its outstanding flux permeation when applied in real seawater.

## Figures and Tables

**Table 1:** Composition of dope solution hollow fiber membranes.

Membrane Designation	PVDF (wt. %)	PEG (wt. %)	DMAc (wt. %)	LSMM (wt. %)	BSMM (wt. %)
PVDF/PEG	18	5	77	0	0
PVDF/PEG/3LSMM-BSMM	18	5	74	3	1
PVDF/PEG/4LSMM-BSMM	18	5	73	4	1
PVDF/PEG/5LSMM-BSMM	18	5	72	5	1

**Table 2:** Composition of dope solution hollow fiber membranes.

Spinning Conditions	Value
Bore Fluid	Distilled Water
OD/ID spinneret size (mm)	1.30/0.55
Air gap (cm)	10
Bore fluid flow rate (mL·min^−1^)	0.6
Gear pump rotation (rpm)	5
Take-up drum rate (rpm)	4

**Table 3:** Properties of membranes with respect to membrane thickness, average pore size, and surface porosity.

Membrane	Thickness (µm)	Mean Pore Diameter (µm)	Surface Porosity (%)
PVDF/PEG	165	0.35	83.50
PVDF/PEG/3LSMM-BSMM	183	0.23	77.70
PVDF/PEG/4LSMM-BSMM	181	0.23	77.27
PVDF/PEG/5LSMM-BSMM	257	0.13	73.93

## Data Availability

Not applicable.
